# New insights into chronic inducible urticaria

**DOI:** 10.1007/s11882-024-01160-y

**Published:** 2024-07-19

**Authors:** Melba Muñoz, Lea Alice Kiefer, Manuel P. Pereira, Mojca Bizjak, Marcus Maurer

**Affiliations:** 1 Institute of Allergology, Charité – Universitätsmedizin Berlin, corporate member of Freie Universität Berlin, Humboldt-Universität Zu Berlin, and Berlin Institute of Health, 10178 Berlin, Germany; 2 Fraunhofer Institute for Translational Medicine and Pharmacology ITMP, Immunology and Allergology Berlin, 12203 Berlin, Germany; 3 Division of Allergy, University Clinic of Respiratory and Allergic Diseases Golnik, Golnik, Slovenia

**Keywords:** Inducible urticaria, Mast cell, Dermographism, Cold, Heat, Delayed pressure, Cholinergic, Solar, Vibratory, Provocation, IgE

## Abstract

**Purpose of Review:**

Chronic inducible urticaria (CIndU) is a group of long-persisting and challenging to manage diseases, characterized by recurrent wheals and angioedema induced by definite triggers. In this review, we address recent findings on CIndU pathogenesis, diagnosis as well as its treatment, and we discuss novel potential targets that may lead to the development of more effective therapies for CIndU patients.

**Recent Advances:**

Meaningful advances in the understanding of its pathogenesis have been reported in the last decades. Novel CIndU-specific patient-reported outcome measures enable a closer and better evaluation of patients.

**Summary:**

CIndU is a hard-to-treat disease that highly impairs quality of life (QoL) of affected patients. Provocation tests allow to diagnose CIndU subtypes. The only licensed and recommended treatment for CIndU are second generation non-sedating H1-antihistamines, which lack efficacy in many cases. Omalizumab off-label use has been assessed in all types of CIndU with overall good outcomes. Promising emerging therapies currently assessed in chronic spontaneous urticaria are paving the path for novel treatments for CIndU

## Introduction

Chronic inducible urticaria (CIndU) is a subgroup of chronic urticaria (CU) characterized by the appearance of recurrent itchy wheals, angioedema, or both as a response to specific, definite, and reproducible triggers. These triggers are physical or chemical, and they include friction, pressure, cold and heat, solar exposure, vibration, activities that induce sweating, contact with urticariogenic substances, and water (Table 1). CIndU is distinct from other forms of CU in that the wheals and angioedema occur only after exposure to these triggers and not spontaneously. The prevalence of CIndU is estimated to be around 0.5% [1]. Recent developments in CIndU include the identification of pathogenic pathways, novel treatments in clinical trials, and the use of patient-reported outcomes for the assessment of CIndU disease activity, impact, and control. Here, we review and discuss recent insights and developments in CIndU, and we highlight the importance of bringing them to routine clinical practice.

**Table 1:** Subtypes of CIndU

**Subtypes of chronic inducible urticaria**		
**Physical urticaria**		**Urticariogenic agent**
Symptomatic dermographism		Shearing forces on the skin (rubbing, stroking, scratching)
Delayed pressure urticaria		Sustained pressure on the skin
Cold urticaria		Exposure to cold
Solar urticaria		Exposure to UV and/or visible light
Heat urticaria		Exposure to heat
Vibratory angioedema		Exposure to vibration

**Other inducible urticaria**		
Cholinergenic urticaria		Active or passive body warming
Contact urticaria		Contact with eliciting agent
Aquagenic urticaria		Exposure to water

## Novel Insights into the Pathogenesis of CIndU

The pathogenesis of CIndU is the focus of several recent and ongoing studies. Pathogenic mechanisms that are involved in chronic spontaneous urticaria (CSU) appear to also play an important role in CIndU [2]. Mast cell (MC) activation and degranulation, together with the subsequent release of histamine and other inflammatory mediators, are the key drivers of CIndU skin lesion development. A recent study confirmed that MCs are indispensable for the development of symptomatic dermographism (SD) and cold urticaria (ColdU). A single treatment with barzolvolimab, an anti-KIT monoclonal antibody (mAb) that depletes MCs, completely abolished symptoms in SD and ColdU patients [3].

Furthermore, it has been hypothesized that autoallergic IgE-mediated MC activation is responsible for the development of the signs and symptoms of SD, ColdU, solar urticaria (SolU), and cholinergic urticaria (CholU) [4–8]. The development of wheals in response to skin friction that characterizes SD was shown to be transferable by serum transfer experiments [6]. It has also been proposed that de novo synthesized autoantigens (autoallergens) can be induced by physical or environmental triggers such as cold, and subsequently detected by IgE bound to skin mast cells (MCs) leading to degranulation in ColdU patients [7]. In SolU, molecular modifications of a putative chromophore by solar electromagnetic radiation might produce IgE-dependent mast cell activation [9]. Passive transfer experiments have also shown the development of wheal reactions after UV exposure in healthy skin previously injected with serum of SolU patients [10]. Autologous sweat and serum induced whealing in CholU patients as well as histamine release from basophils suggest that some patients with CholU may have a type I (IgE-mediated) allergy to their own sweat [11].

Overexpression of the IgE receptor, FcεRI, on basophils is observed in patients with CIndU independently of the subtype and is comparable to the expression found in CSU patients [12]. In addition, basophil activation was increased at steady state in patients with CIndU compared to that of healthy controls [13]. Altogether, this evidence indicates that skin MC and IgE play an important role in the pathogenesis of CIndU. Moreover, there is direct and indirect evidence of histamine release by in vivo and in vitro analyses in all types of CIndU [14].

## Novel CIndU-specific Clinical Insights and Developments

### Physical Urticaria

#### Symptomatic Dermographism

SD, formerly also known as urticaria factitia, is the most common subtype of CIndU [15]. Itchy and strip-shaped wheals commonly develop approximately 1 to 5 min after shearing forces on the skin are applied such as stroking, scratching, scrubbing, or rubbing [16]. The wheals and itch usually last for around 30 min; however; they can occur very frequently resulting in strong impairment of patients’ (QoL) [17]. Recent studies showed that the gut microbiome of SD patients is imbalanced: beneficial bacteria, mainly short chain fatty acid producing bacteria such as *Verrucomicrobia phylum* and *Ruminococcaceae* family as well as the alpha diversity are decreased, whereas conditional pathogenic bacteria such as *Enterobacteriales* order are increased in SD patients [18]. *Subdoligranulum* and *Ruminococcus bromii* were suggested as promising diagnostic biomarkers of SD [19].

In addition, other SD biomarkers have been postulated. Two microRNAs, miR-126-3p and miR-16-5p, were significantly downregulated in patients with active SD but upregulated when patients were in remission [20]. Interestingly, these two miRNAs target vascular endothelial growth factor -A, which was significantly increased in active SD patients and decreased in remission [20]. Thus, vascular endothelial growth factor-A levels may reflect disease activity and treatment response of SD patients.

Recently, new SD variants have been reported such as the food-exacerbated SD, where lower trigger thresholds are observed after food intake, and food-dependent SD, where positive provocation test reactions appear only after the intake of food [21, 22]. Interestingly, physical exercise can also impact disease activity in SD patients. In a recent study, reduced skin provocation test responses were observed in 83% of SD patients after reaching a target heart rate of 140 to 170 beats/min [22]. Thus, eating appears to increase SD disease activity whereas exercising decreases disease activity in some SD patients.

#### Delayed Pressure Urticaria

Patients with delayed pressure urticaria (DPU) develop swellings after 4 to 6 h of skin exposure to a sustained pressure stimulus, although in some cases, a delay of 12 to 24 h for the appearance of the lesions is observed [23]. Symptom burden together with the limitation on daily activities and clothing cause a strong impact on QoL in DPU patients compared to other forms of CIndU [23]. A recent study that evaluated DPU patients who received omalizumab showed that 75% of DPU patients exhibit some characteristics of an autoallergic endotype such as normal IgG anti-TPO levels and total IgE > 100 IU/mL [24]. In addition, CRP > 0.5 mg/dL was observed in 43% and D-dimer levels were > 500 ng/mL in 30% of DPU patients [24]. DPU patients in this study responded well and fast to omalizumab, consistent with the proposed autoallergic endotype of most of these patients.

#### Cold Urticaria

ColdU is characterized by wheals, angioedema, or both, occurring rapidly in response to cooling [25]. Its estimated annual incidence is 0.05% [26]. The diagnosis of typical ColdU is based on immediate whealing in response to a standard provocation test. In atypical forms, unusual responses are seen (e.g., delayed whealing), or specific provocation methods (e.g., total body cooling) are required [27]. Patients with ColdU may react to various cold triggers, including exposure to cold air, contact with fluids or solid surfaces, and the ingestion of cold foods or drinks [25, 28]. There can be a considerable heterogeneity in clinical presentations, ranging from mild localized whealing to cold-induced anaphylaxis (ColdA) [25, 29].

ColdA, a potentially life-threatening systemic reaction, requires adrenaline administration but is frequently undertreated [30]. Its fatality rate remains unknown [27]. ColdA has recently been included in the list of indications for prescribing adrenaline (epinephrine) autoinjector [31]. Over the past four decades, several studies have reported systemic reactions in ColdU, with a varied incidence ranging from 4 to 47% of ColdU patients [25]. The pathogenesis of ColdA is not completely understood. Recent findings from the multicenter COLD-CE study show that patients with ColdA are more likely to experience cold-induced angioedema, oropharyngeal/laryngeal symptoms, and itchy earlobes compared to ColdU patients without systemic manifestations. The primary factors influencing systemic reactions appear to be the exposed skin’s surface area, temperature, and duration of exposure. Aquatic activity is the most common trigger, rather than cold weather or localized contact with cold fluids or objects [25, 32]. This suggests a dose dependency, which is not characteristic of classical IgE-mediated anaphylaxis [27]. ColdU is mostly acquired; however, rare hereditary autoinflammatory diseases that are interleukin-1 and factor XII– mediated have been reported. These syndromes are characterized by a cold-induced urticarial rash and systemic symptoms, including recurrent fever, arthralgia, and fatigue [33, 34].

#### Solar Urticaria

SolU is defined by the appearance of erythema, itch and the formation of wheals within minutes of exposure to light, mostly sunlight, the components of solar radiation, but also to artificial light sources [15, 35]. SolU accounts for less than 0.5% of all CU cases and makes for 0.7% and 3.2% of all photodermatoses in black and white patients, respectively [36]. IgE has been suggested as a possible serum factor responsible for the development of SolU symptoms; however, the only current evidence in support of this hypothesis relies on the efficacy of omalizumab treatment in some SolU cases [37, 38]. Novel diagnostic methods using a basophil activation test with irradiated patient’s serum to assess serum photoallergens have been reported [39]. Such alternative approaches might be helpful in cases where light provocation tests are negative.

#### Heat Urticaria

Heat urticaria is a very rare form of CIndU, defined by the appearance of wheals and itch after contact with heat or a hot object within minutes of exposure [15]. Case reports showing good efficacy of treatment with omalizumab suggest that IgE and the FcER1 may be involved in its pathogenesis [40].

#### Vibratory Angioedema

Vibratory angioedema is a very rare CIndU characterized by the occurrence of erythematous wheals and angioedema immediately or within 10 min after exposure to vibration at contact sites [15]. Hereditary vibratory angioedema has been associated with a gain-of-function mutation in the adhesion G protein- coupled receptor E2, located on MCs, which might decrease the inhibitory interaction between the α- subunit and the β- subunit of this receptor, leading to sensitization of MCs to vibration-induced degranulation [41]. A systematic review of vibratory angioedema studies showed that whealing in response to exposure to vibration was observed more frequently in patients with hereditary vibratory angioedema whereas angioedema was found more frequently in acquired vibratory angioedema[41].

## Other Inducible Urticaria

### Cholinergic Urticaria

CholU is characterized by the recurrent appearance of itchy wheals and/or angioedema induced by exercise and passive warming [15]. Emotional stress as well as spicy and hot food can also elicit responses. CholU patients typically exhibit tiny, short-lived wheals with a pronounced flare mainly on the upper trunk and extremities and lasting 15–60 min [42]. CholU predominantly affects young men, with prevalence studies indicating up to 20% of young students showing CholU symptoms [43]. CholU is frequently associated with atopic conditions [44] and should be differentiated from exercise-induced anaphylaxis [45]. CholU patients with anhidrosis often experience worsening in colder living environments with exposure to temperature differences. Four subtypes of CholU based on the pathogenesis and clinical characteristics have been proposed: (i) conventional sweat allergy-type CholU, (ii) follicular-type CholU with a positive autologous serum skin test, (iii) CholU with palpebral angioedema, and (iv) CholU with acquired anhidrosis and/or hypohidrosis [46].

Concentrations of specific IgE to the sweat antigen MGL_1304 of Malassezia globosa, a major allergen in human sweat of patients with atopic dermatitis, were significantly higher in sera of CholU patients compared to normal controls [47]. CholU patients with positive autologous serum skin test and no sweat allergy showed erythematous wheals associated with hair follicles. In addition to autologous serum skin test, acetylcholine (Ach) overflow that cannot be trapped by its CHRM3 receptor might activate hair follicle-adjacent MCs to produce wheals [48]. In contrast, CholU can be associated with acquired idiopathic generalized anhidrosis where blockade of the sweat gland ducts may cause sweat reflux containing histamine or a sweat antigen that could lead to the symptoms [49].

Furthermore, sweat glands of CholU patients exhibited significantly reduced CHRM3 and Ach esterase expression, particularly in those who exhibit anhidrosis [50]. Interestingly, CholU patients with severely impaired sweating showed long disease persistence and higher disease severity [50]. Even though the sensitivity to identify CholU through Ach-prick testing is low, ACh-induced wheals, in patients with CholU, are linked to sweating and longer lasting symptoms [51].

### Contact Urticaria

Contact urticaria presents with the development of urticarial lesions within minutes, usually within 30 min after direct contact with exogenous agents. Contact urticaria can be nonimmunological, where the response is localized at the site of contact with an eliciting agent, usually plants or chemicals. In addition, contact urticaria can be immunological, characterized by a reaction that is not only localized but also spreads due to an IgE-mediated reaction either to a high molecular weight protein or hapten chemicals of low molecular weight [52, 53]. No real-world data are available on the prevalence of contact urticaria in the general population.

### Aquagenic Urticaria

Aquagenic urticaria is a rare variant of CIndU characterized by the occurrence of itchy wheals after skin contact with water [15]. Typically, aquagenic urticaria patients develop small wheals within 30 min after water exposure that last for 30 to 60 min [15, 54]. Aquagenic urticaria is induced by the exposure to water regardless of its temperature, and it has been hypothesized that a MC-degranulating substance results from the interaction between water and sebum [55]. Salinity is an important factor for some patients who show reactions only in contact with seawater [56]. Some other factors that might be involved in the pathogenesis of aquagenic urticaria are changes in osmotic pressure surrounding hair follicles with increased passive diffusion of water or histamine-independent unknown mechanisms [57].

A systematic review of the literature on aquagenic urticaria revealed that the most common trigger is tap water and the most frequent affected part of the body is the trunk [57]. Saline or seawater induce lesions mostly in the neck, face and submandibular skin [56]. It is important to discriminate between aquagenic urticaria and aquagenic pruritus, which is characterized by itch without visible skin changes after contact with water [58], since aquagenic pruritus, in some cases, is associated with lymphoproliferative disorders such as polycythemia vera and other myelodysplastic syndromes [59].

## New Developments in CIndU Diagnostic Workup and Monitoring

**Fig. 1:**
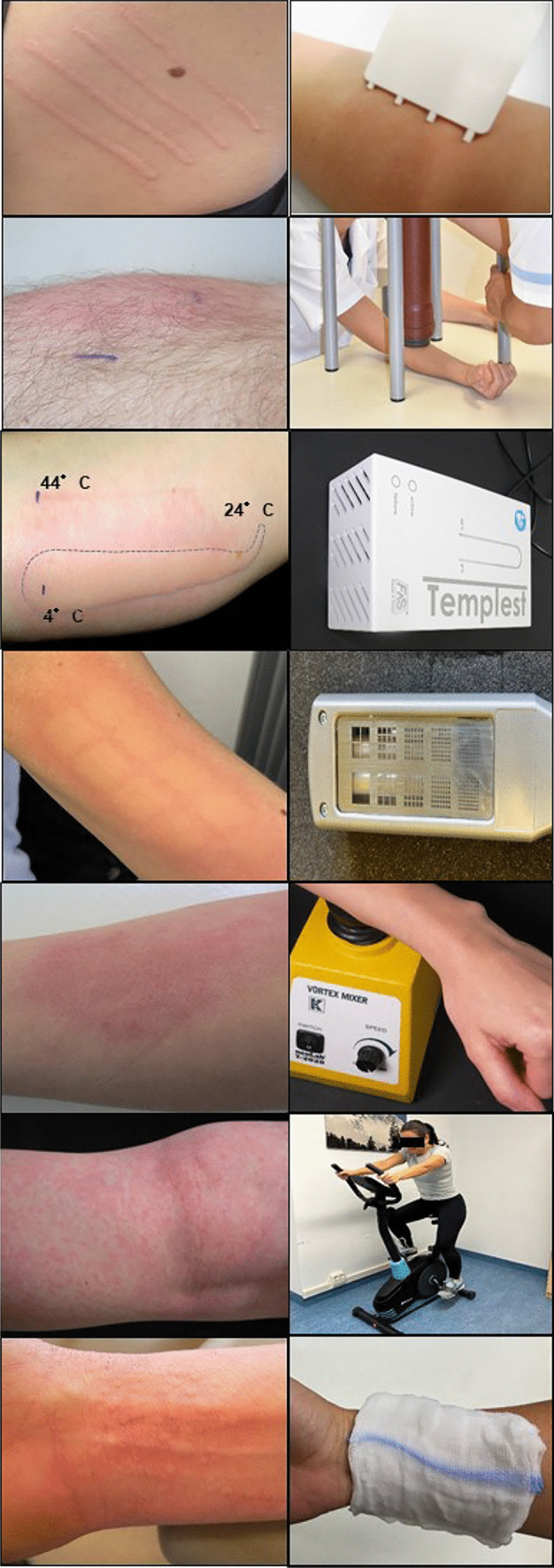
Provocation tests for CIndU diagnosis

The diagnosis of CIndU is based on a thorough medical history and specific provocation testing (Table 2, Fig. 1) [60, 61]. The aim of the diagnostic work-up is to confirm the diagnosis, determine relevant triggers, assess trigger thresholds, and establish starting points (baseline) for monitoring treatment response. Provocation testing should be performed and assessed by a trained physician in standardized settings, and emergency treatment should be available since some CIndU forms (ColdU, CholU, SolU, heat urticaria, contact urticaria) bear a certain risk of systemic reactions. Results of the provocation testing can be influenced by several factors, including exercise, recent trigger exposure, and concomitant treatment (e.g. with antihistamines). Skin sensitivity to eliciting triggers is assessed by provocation testing, which is useful for counseling patients on how to avoid triggers, assessing treatment responses, and improving disease management [60].

**Table 2:** Provocation testing overview

Diagnosis	Provocation Test	Test localization/ Exposure time	Time of assessment of reaction
Symptomatic dermographism	^1^FricTest® ^2^Dermographometer	Volar forearm or back Seconds during stroking	Within 10 min
Delayed pressure urticaria	Weight rod suspension	7 kg over the shoulder 5 kg over the volar forearm 15 min	From 4–6 h on
Cold urticaria	^3^TempTest® Melting ice cube (Plastic bag)	Volar Forearm 5 min	Within 10 min
Solar urticaria	^4^UVA/UVB/VL light source	Buttocks Volar upper arms 5 min	Within 10 min
Heat urticaria	^3^TempTest®	Volar Forearm 5 min	Within 10 min
Vibratory angioedema	^5^Vortex mixer	Volar Forearm 5 min	Within 10 min
Cholinergic urticaria	^6^Pulse controlled ergometry	Exercise on a static bicycle for 30 min	Within 10 min
Contact urticaria	Eliciting agent, specific IgE	Volar Arm or known affected area 1–2 min	Within minutes, (in some cases 30 min)
Aquagenic urticaria	Wet towel (soaked with 35–37 °C water) placed on the skin	Trunk or known affected area 5 min	Within 10 min

^1^ FricTest® (Moxie GmbH, Berlin Germany) [62]

^2^ Calibrated dermographometer with scale settings from 0 to 15, equivalent to a range of tip pressures from approximately 20 to 160 g/mm^2^, (HTZ Limited, New Addington, UK) [15]

^3^TempTest®: with a temperature gradient ranging from 4 to 44 °C (Courage & Khazaka, Köln, Germany) [63]

^4^Hand-held UV-A and/or UV-B device. If available, solar simulators with filters (UV-A and UV-B) or monochromator (UV-A and UV-B, visible light)

^5^Laboratory vortex mixer (mixer runs 780 rpm to 1380 rpm for 5 min) [15]

^6^Bicycle ergometer (an increase in pulse rate of 15 beats every 5 min to a final maximum increase of 90 beats per minute during 30 min) [64]. Pre-existing cardiac problems, should be ruled out prior to provocation

Validated disease-specific patient-reported outcomes should be used to assess disease activity, impact on QoL, and disease control in all CIndU patients at the first and all subsequent visits in the clinical practice. Patient-reported outcomes enable the recording of the unfiltered patient perspective, preventing the loss of important information that may occur with isolated evaluations by physicians. The CholU-QoL, a 28-item disease-specific validated questionnaire, is used to assess overall CholU-related QoL impairment [65]. Additional CIndU patient-reported outcomes that assess disease-specific QoL impairment, such as the ColdU-QoL and SD-QoL, are available but not yet validated. CIndU patient-reported outcomes that evaluate disease activity, i.e. the CholUAS, ColdUAS and SDAS, are also available but not yet validated. In all CIndUs, disease control can and should be assessed by the urticaria control test (UCT) [66].

## Novel Insights on the Management and Treatment of CIndU

Different treatment schemes have been used in the management of CIndUs. Avoidance of known triggers is advised to control symptoms; however; this approach can be very challenging and it is associated with high impairment of QoL. Therefore, treatment that prevents the development of signs and symptoms has to be implemented in most cases. sgAHs have been widely used in the management of CIndU [15]. However, a high percentage of CIndU patients do not take any sgAH as revealed in the AWARE study, where only 1 in 5 patients with CIndU was treated with sgAH [67]. Beyond sgAHs, there are currently no licensed drugs indicated for CIndU. Omalizumab can be used as an alternative therapy, but its use is off-label in patients with standalone CIndU [15]. Advances in understanding the pathogenesis of CSU have allowed the emergence of novel drugs that specifically target mediators, receptors or signaling pathways involved in the activation or inhibition of MC and other immune cells. Several of these novel therapies have also been and will be evaluated in CIndU patients.

## Antihistamines

Daily intake of sgAHs at the licensed dose is the first-line therapy advised for all CIndUs. Patients who do not achieve complete control with the licensed dose should increase their antihistamine dose up to 4 times the standard daily dose [61]. Several studies that have evaluated the response of CIndU patients to standard and updosing of sgAH showed different outcomes when comparing CIndU and CSU. One report showed that only one fifth of CIndU patients (20%) responded to standard doses of sgAHs [68]. However, a recent study showed that isolated CIndU patients were less refractory to sgAH than CSU patients and CSU with accompanying CIndU (19.8% vs 29.7 and 39% respectively) [69]. In contrast, another report showed that CIndU patients with and without comorbid CSU show similar rates of response to treatment with sgAHs [70]. In a pedriatic cohort of CIndU patients, the majority (72%) were controlled with a standard dose of sgAH and 16% required updosing of sgAH [71]. From these patients, 18% of ColdU, 50% of DPU, and 19% CholU required twice the standard dose and 3% of ColdU patients required 4 times sgAHs [71].

A systematic review of treatments in SD revealed that first-generation H1-antihistamines were the most frequently studied therapy and showed variable efficacy and significant side effects [72]. In contrast, sgAH were effective and well tolerated [72]. However, most SD studies were heterogeneous, monocentric, and included a small number of cases, making difficult to draw conclusions. Similarly, a systematic review of treatment options for DPU showed that sgAHs remain the first-line therapy due to the lack of other evidence and the scarcity of studies on DPU [23]. Furthermore, another review of eight independent studies that evaluated sgAHs in the therapy of ColdU demonstrated that higher-than-standard doses are more effective than standard doses, and that updosed sgAHs do not increase the risk of adverse events [73]. Several sgAHs, e.g. bilastine, desloratadine, ebastine, and rupatadine, were shown to significantly inhibit wheal formation after cold provocation compared to placebo [74–77]. The proportion of CholU patients who benefited from standard-dosed sgAH treatment is usually low. In one study, updosing of sgAH resulted in relatively low response to sgAH (38%) [78]. Thus, overall, sgAHs are generally, but not always effective, and available data indicate that updosing works when standard doses do not. However, the supporting evidence is limited by small studies with few patients, poor reporting quality and heterogeneous outcomes.

## Anti-IgE Therapy

Omalizumab is a humanized anti-IgE mAb that is very effective in antihistamine-refractory CSU patients and has been licensed for CSU since 2014 [79]. Omalizumab has also been shown to be effective in CIndU [8, 80]. A randomized, placebo-controlled trial involving 55 patients with SD demonstrated the efficacy of omalizumab 150 mg and 300 mg in improving friction trigger thresholds, as early as week 4 after the start of treatment [81]. In a recent report, SD patients showed a significant increase in UCT scores from 4.7 ± 2.3 to 14 ± 2.9 with omalizumab treatment [82]. Omalizumab also appears to be rapidly effective and well tolerated in patients with ColdU. A randomized, placebo-controlled trial including 31 ColdU patients demonstrated significant clinical superiority of omalizumab versus placebo [83]. A recent study also showed a good response to omalizumab at a dose of 300 mg every 4 weeks among 9 ColdU patients, with five patients achieving complete response [84]. Data on the use of omalizumab in children are limited, but a few studies have assessed its efficacy. One report showed that 6 of 13 ColdU children (4–16 years) achieved a complete response to omalizumab, and 6 achieved a partial response [85].

Omalizumab has also been evaluated in DPU patients. Good efficacy was observed in most cases, with only 4 treatment failures reported [8, 86–93]. Furthermore, another recent study assessed omalizumab responses in 14 DPU patients. Median UCT levels before treatment (3.1 ± 2.4) markedly increased (15.3 ± 1.7) after the first dose of omalizumab [24]. None of the patients experienced an adverse reaction [24].

Omalizumab can also be a beneficial and well-tolerated long-term therapy for SolU. In several publications with a total of 36 cases, omalizumab showed efficacy in symptom control in 18 patients [8, 94], with 8 patients achieving a partial response [89, 90, 95, 96] and 8 not responding to treatment [86, 97, 98]. In a recent retrospective study in Spain, complete response, as assessed by UCT and Urticaria Activity Score over 7 days, was achieved and maintained in 90% (18/20) of SolU cases [38]. There is evidence that omalizumab can be an effective treatment option for heat urticaria as well. Five reports have shown that it led to marked clinical improvement in a total of 6 patients, where doses between 300 and 450 mg every 2 and 4 weeks were used [40, 88, 89, 99, 100].

Responses to omalizumab therapy in CholU patients have been shown to be less beneficial compared to other subtypes of CIndU. A multicenter randomized placebo-controlled trial in Spain found no statistically significant difference between 13 patients treated with omalizumab and 9 patients treated with placebo [101]. In a prospective study in Germany, 3 of 6 CholU patients exhibited a complete response to omalizumab, while 2 patients displayed minor improvement, and one patient showed no response [78]. A delayed response to omalizumab in CholU patients has also been observed, where increasing the dose is required to achieve a significant response [84].

Only a few reports assessing the efficacy of omalizumab in aquagenic urticaria are found in the literature. One case of a patient with salt-dependent aquagenic urticaria showed a complete response to omalizumab 300 mg every 4 weeks [102]. Another recent case also demonstrated a full response after the initiation of omalizumab [103], while another case achieved complete response after 2 months of treatment [104]. Thus, there is evidence that omalizumab can be effective in the treatment of CIndU, and this efficacy seems to depend on the dose and the subtype of CIndU. However, data are limited to case reports, case series, or small randomized controlled trials and the efficacy appears to be somewhat lower compared to that in CSU.

## Alternative Treatment Options

Additional therapy options with lower evidence have been used in the treatment of some forms of CIndU. Case series and reports showed efficacy of antibiotics such as penicillin or doxycycline in the treatment of ColdU [105, 106]. The mechanisms leading to the amelioration of symptoms by these medications in ColdU remain largely unknown, and controlled studies are needed to confirm their efficacy. In addition, desensitization to eliciting triggers has also been assessed in some CIndU patients, particularly for ColdU, heat urticaria and SolU [107–109]. Skin desensitization to cold can also be achieved by repeated cold exposure [107, 110]. However, this treatment option poses a risk for anaphylaxis during induction and requires daily cold showers to maintain the protective effect that may be affected by the lack of patient compliance. Similarly, desensitization protocols for CholU treatment involving regular physical exercise or hot baths have been described in some patients. Repeated sweating could be effective in CholU patients with sweat allergy and can also prevent poral occlusion [111]. Furthermore, tolerance can be induced by repetitive exposure to UV radiation of increasing doses, such as UVA, a process known as phototherapy hardening [112]. Although this method seems effective, it is time-consuming, and long-term phototherapy is usually required to maintain tolerance. Intravenous immunoglobulins have been considered as a treatment option for SolU; however, a single course of intravenous immunoglobulins seems to be insufficient to obtain sustained disease control [113].

## Novel CIndU Treatment Options Under Development

Dupilumab, a mAb against the interleukin-4 receptor alpha subunit (IL-4Rα), inhibits the signaling pathways of interleukin-4 (IL-4) and interleukin-13 (IL-13) [114]. Dupilumab reduced disease activity in mAb naïve patients with sgAH-refractory CSU and was well tolerated [115]. A case report showed good efficacy in a SolU patient who was omalizumab resistant and achieved well controlled disease at week 16 of dupilumab treatment [116]. Moreover, dupilumab efficacy has also been shown for ColdU. Two recent reports showed that a 38-year-old patient exhibited a rapid complete disappearance of her symptoms, without any flare-up during cold exposure [117] and a 28-year-old patient with atopic dermatitis associated with ColdU also achieved complete control after treatment with dupilumab [118]. In addition, a 26-year-old man suffering from CholU was also successfully treated with dupilumab [119]. Phase II and phase III randomized clinical trials investigating its efficacy in CholU (NCT03749148) are ongoing; however, the phase III study in ColdU did not meet the required efficacy endpoints [2].

Benralizumab is an anti-α chain of the IL-5 receptor mAb that leads to eosinophil depletion [120]. A case report showed that a patient with severe SD benefitted from benralizumab highlighting a potential role of IL-5 in CIndU [121]. Reslizumab, an anti-IL-5 mAb licensed for the treatment of severe eosinophilic asthma, was effective in the treatment of a patient who had not only asthma but also CSU and ColdU [122]. Lirentelimab binds to sialic acid–binding immunoglobulin-like lectin-8, an inhibitory receptor on eosinophils and MCs, thereby depleting eosinophils via apoptosis and inhibiting MC activation. Lirentelimab led to improved disease control in CholU and SD patients shown by increase in UCT score compared to the baseline [123]. There are currently no controlled trials evaluating lirentelimab in CIndU.

Bruton´s tyrosine kinase (BTK) is a cytoplasmatic tyrosine kinase expressed in various immune cell populations including B cells, MCs, basophils, macrophages and platelets [124]. As BTK is localized downstream of the FcεR1, it constitutes a promising target for MC-associated diseases [125]. BTK inhibition leads to a decrease in histamine release and production of pro-inflammatory mediators by MCs and basophils [126, 127]. Remibrutinib, a highly selective oral BTK inhibitor, has shown substantial efficacy and a favorable safety profile in phase IIb and phase III trials enrolling patients with CSU [128–130]. Efficacy data are still missing regarding the use of BTK inhibitors in CIndU. A phase III randomized clinical trials investigating remibrutinib efficacy in patients with SD, ColdU and CholU will be conducted soon (NCT05976243). Moreover, rilzabrutinib is currently being investigated for CSU in a phase II trial (NCT05107115).

Novel mAbs that target IgE or its receptor have been considered as potential treatments for CIndU. The IgE-Trap protein (YH35324) with a high affinity for serum free IgE is currently under investigation in patients with ColdU in a phase I trial (NCT05960708).

Barzolvolimab (CDX-0159) is a humanized immunoglobulin G1 kappa mAb that binds the extracellular domain of KIT with high specificity [131]. A single intravenous dose of barzolvolimab significantly reduced the numbers of skin MCs leading to a marked decrease of disease activity in SD and ColdU patients [3]. In addition, this study underlines the central role of skin MCs and KIT/SCF signaling in ColdU and SD. Apart from hair color changes and selective taste changes for umami and salty flavors, barzolvolimab was well tolerated. Phase II studies in CIndU, are currently ongoing. Additional anti-KIT antibodies such as briquilimab will also be evaluated in CIndU.

Another treatment in development for CIndU patients targets Mas-Related G Protein-Coupled Receptor X2 (MRGPRX2), which is responsible for IgE-independent activation of MCs. Patients with severe CSU have increased numbers of MRGPRX2-expressing MCs in lesional skin [132]. An oral MRGPRX2 antagonist, EP262 is currently under investigation in SD and ColdU (NCT06050928). At least one more MRGPRX2 antagonist, EVO756 is in preclinical development. Lastly, H-4 receptor (H4R) antagonists including JNJ7777120, JNJ39758979, INCB38579 have shown to reduce pruritus in humans [133] and may be potential targets in treating CIndU patients.

## Unmet Needs in CIndU

Current unmet needs in CIndU include: 1. Lack of effective treatment options: Many patients with CIndU do not respond adequately to antihistamine treatment. The need for alternative treatment options is addressed, to some extent, by ongoing studies of novel therapies. However, further programs are needed to develop disease-modifying treatments and, ultimately, curative therapies. 2. Disease burden and impact on QoL: CIndU can significantly impact a patient's QoL, but further studies are needed to identify drivers of QoL impairment and the burden associated with CIndU. 3. Understanding of disease mechanisms: Further research is needed to better understand the underlying mechanisms of CIndU, including the molecular triggers of MC degranulation and the upstream pathophysiological processes that lead to their generation. A better understanding of the pathogenesis can lead to the development of targeted therapies. 4. Patient education and support: Patients with CIndU benefit from increased awareness and education about their condition, as well as access to support networks and resources to help them manage their symptoms.

## Conclusions

CIndU is a hard-to-treat and long lasting skin disorder that highly impairs QoL of affected patients. Provocation tests allow to diagnose CIndU subtypes and patient-reported outcomes help to assess treatment responses in the clinical practice. The efficacy of the emergent therapies in CIndU will shed light on the cellular and molecular mechanisms underlying the pathogenesis of CIndU.

## Data Availability

No datasets were generated or analysed during the current study.

## Abbreviations

Ach: Acetylcholine
BTK: Bruton’s tyrosine kinase
CholU: Cholinergic urticaria
CIndU: Chronic inducible urticaria
ColdA: Cold-induced anaphylaxis
ColdU: Cold urticaria
CSU: Chronic spontaneous urticaria
CU: Chronic urticaria
DPU: Delayed pressure urticaria
mAb: Monoclonal antibodies
MC: Mast cell(s)
MRGPRX2: Mas-Related G Protein-Coupled Receptor X2
QoL: Quality of life
RCT: Randomized controlled trial
SD: Symptomatic dermographism
sgAH: Second-generation H1-antihistamine
SolU: Solar urticaria
UCT: Urticaria control test
